# Human Health; or the Influence of Atmosphere and Locality; Change of Air and Climate; Seasons; Food; Clothing; Bathing and Mineral Springs; Exercise; Sleep; Corporeal and Intellectual Pursuits, &c. &c. on Healthy Man, Constituting Elements of Hygiene

**Published:** 1845-10

**Authors:** 


Art. II.—*Human Health; or the Influence of Atmosphere and Locality; Change of Air and Climate; Seasons; Food; Clothing; Bathing and Mineral Springs; Exercise; Sleep; Corporeal and Intellectual Pursuits, &c. &c. on Healthy Man, constituting Elements of Hygiene.* By Robley Dunglison, m.d. &c.—*Philadelphia,* 1844. 8vo, pp. 464.

There is nothing in this work calling for lengthened notice, but in saying so, we are far indeed from intending to convey a mean opinion of it. There is nothing essentially novel in the plan or materials of the work. In its general features, arrangement and execution, it resembles treatises on the same subject already before the public. We therefore do not consider it necessary, as we have just observed, to analyse it in detail. But we can safely recommend it as a volume comprising a great deal of useful and entertaining information, though this consists principally of compilation not of original research. The portion of the work allotted to the consideration of "food," and which occupies nearly a third of the volume, if it does not contain much scientific investigation, method, or remark, exhibits a good deal of practical observation, and being popular in its language and explanations, might form part in a system of *domestic medicine,* superior to any at present before the public. The work, in fact, is quite as well adapted for the general as for the professional reader; nay, we should say, that with more than usual felicity, it is fitted for both classes of readers; it is a treatise, which the scientific man and the physician will read without contempt; the ordinary reader, without difficulty and with equal pleasure and improvement.

